# Looking to the Future of Work and Family Theory and Research: Some Reflections

**DOI:** 10.1111/jftr.12198

**Published:** 2017-06-01

**Authors:** Oriel Sullivan

**Affiliations:** ^1^ Oxford University

**Keywords:** Family and work

Maureen Perry‐Jenkins and Shelley MacDermid Wadsworth (2017) have provided a thought‐provoking review of some of the topics and theoretical themes that have inspired researchers in the area of work and family research from the start of the 21st century. They had the interesting idea of presenting an overview of nominations for the Kanter Award since the year 2000, seen through a specific theoretical lens—the ecological perspective. I should state from the outset that, belonging to a different disciplinary context, I am not overly familiar with the ecological perspective. What follows is a series of reflections, from the perspective of a European sociologist, that relate in direct or less direct ways to the arguments presented in the final section of the article, “Looking to the Future of Work and Family Theory and Research.” I say more below, however, about our disciplinary differences, because it seems to me that there may be some interesting interdisciplinary connections between approaches that I am more familiar with and the perspective taken by the authors.

Taking the items in the final section of the article— “Looking to the Future”—in the order in which they appear in the article, I am completely in agreement with the call for more attention to be given to conceptual clarity in the ongoing exploration of the changing meanings of work and family. In particular, I would like to flag a relevant debate currently going on around the meaning of unemployment. Feminist sociologists have devoted much attention to emphasizing the variety of different configurations of family—such that the term itself is commonly substituted with *families*, deliberately signaling diversity. Equally, we have given much effort to defining and understanding the meanings and economic importance of unpaid domestic work and care. Perhaps rather less attention has been given to a consideration of how we conceive of the employment side of work, an area that has changed rapidly over the past few decades (particularly since the 2008 financial crisis). Specifically, we have perhaps been satisfied with too lazy a conception of the terms *employed* and *unemployed* for the current socioeconomic context.

Many sociologists will be familiar with the story of Marienthal, a small factory‐based community outside Vienna, made famous by the 1933 book *Marienthal: A Sociography of an Unemployed Community* (Jahoda, Lazarsfeld, & Zeisl, 1971). The finely grained, multimethod documentation of a “weary” unemployed community countered the then‐current popular idea that the unemployed could be regarded as potential revolutionaries. The book became a classic of its time, but it related to a specific historical conjuncture associated with the demise of traditional textile manufacturing industries during the 1930s Great Depression. The male population of Marienthal was fully employed by a large textile factory, which closed in 1930, creating immediate mass unemployment. The transition from full employment to complete nonemployment provided the background for study of the unemployed of Marienthal.

But postrecession societies are increasingly characterized by a rather different configuration of what unemployment means. In particular, we have seen the rapid growth of “precarious” forms of employment—as indicated by short and unpredictable working hours, the erosion of employment rights, employer‐determined flexibility, and zero‐hour‐contract employment. Our familiar conceptions of what it means to be employed and unemployed in this context are in need of further consideration, as the boundaries become more blurred. Increasingly, those who we include within the “employed” category may resemble those who are unemployed, in terms of the sociopsychological effects of employment precariousness and the impact that this is likely to have on the processes of family relationships.

Turning to the section on methodological innovations, I would like to support and underscore the emphasis on the importance of life‐course analysis—in relation, for example, to the generation of wage penalties for women. As a result of constraints of funding and administration there are at the international level relatively few large‐scale nationally representative household panel studies that provide longitudinal household level data—so important for the assessment of work and family processes and outcomes at the large scale. In the United States there is, of course, the Panel Study of Income Dynamics (1968–) and the Survey of Income and Program Participation (1984–). Australia has the more recently started Household, Income, and Labour Dynamics in Australia Survey (2001–). In Europe we are also relatively well provided for with the German Socio‐Economic Panel (1984–) and the U.K. British Household Panel Survey (1991–), more recently incorporated into the U.K. Household Longitudinal Study (2010–). Because I am more familiar with data about the United Kingdom than elsewhere, I would also like to flag the relevance of a growing series of national‐level cohort studies in the United Kingdom (equivalent to the U.S. National Longitudinal Study of Youth). These began with the 1958 National Child Development Survey and include two additional cohorts born in 1970 and 1990, respectively, with the latest addition being the Millennium Cohort study of children born in the year 2000. These types of data provide a view on the timings and processes underlying the cross‐sectional associations that are frequently the subject of our research findings. They also increasingly collect psychosocial, health, and genetic markers and outcomes that can help us incorporate into our research the more holistic approach to the development of social phenomena which I understand the ecological perspective to be about. Some of these studies have also had time‐use diaries attached to them (the British Cohort Study of 1970, the Millennium Cohort Study of 2000, and the Innovation Panel of the U.K. Household Longitudinal Study), thus offering a combination of time‐use and panel data information.

I would like to illustrate here the potential contribution of life course analyses with a few recent examples of new and relevant research using longitudinal panel data that may not be familiar to U.S. audiences. Laura Langer (2015) and Marta Seiz (2014) both used the German Socio‐Economic Panel, which by now provides researchers with a continuous measure of working hours across several decades of couples' lives, enabling the analysis of long‐term specialization patterns in paid and unpaid labor and care. Langer (2015) argued that much research on the gender division of labor in heterosexual couples has focused mainly on transitions in and out of employment made during the first years of parenthood. A common conclusion has been that couples specialize—women in unpaid and men in paid work—in response to parenthood. But what happens further down the road for these couples? She analyzed the dual‐career trajectories of West German couples from the 1956–1965 female birth cohort, who were young adults at a time when there was still relatively low institutional and normative support for female employment. She found that even in this setting, and at a conservative estimate, a surprisingly small number of couples—only one‐fifth—adopted full specialization in later life, whereas a full third moved into (or back into) dual, full‐time employment. This trend is even more pronounced among highly educated couples: Half of those couples moved into dual full‐time employment (Langer, 2015). A longitudinal perspective, then, informs us that the specialization that might occur around childbearing ages is in many cases reversed later in the life course. Marta Seiz's (2014) thesis also adopted a dual‐trajectory approach to determine the nature of the relationship between men's housework and child‐care participation and women's employment and economic position. She analyzed both the influence of male domestic effort on the female partners' propensity to exit the labor market or leave full‐time work after marriage and childbirth, and whether fathers' domestic inputs affect mothers' earnings trajectories in the long run. She showed that men's domestic participation makes it easier for their female partners to maintain a strong position in the labor market and also serves to reduce the economic penalty experienced after motherhood (Seiz, 2014).

In relation to the call made in the article for more specifically cross‐national perspectives, it does strike me that, although admittedly over recent years several Kanter Awards have gone to articles published in European journals, the fact that the committee selecting the nominations is heavily weighted toward U.S. universities may mean that there is less of an emphasis on cross‐national perspectives than there would have been, perhaps, in a similar collection of research articles written and published in Europe. This effect may be partly structural: Funding from the European Union is very often conditional on cross‐national comparative research undertaken by consortia of researchers across various European Universities and institutions. But as an author publishing in U.S.‐based journals, I frequently find that I am asked by reviewers to provide a “comparative context” when I use only British data, whereas I suspect that authors focusing solely on U.S. data are less frequently asked to do the same! This may also contribute to a stronger focus on cross‐national comparison in research emanating from countries other than the United States.

Having said that, it is clear that, during the 2000s, the research focus has become more cross‐national—in particular, newly developed multilevel methodologies have offered the opportunity to examine the contribution and articulation of macro‐level (e.g., policy, ideology) and micro‐level (e.g., individual education, employment status) variables across a range of countries. For example, research using multilevel analyses has documented the importance of relevant policies, ideological, and institutional structures underpinning (a) time spent in paid and unpaid work, (b) the domestic division of labor, (c) men's unpaid work and care, and (d) women's employment and education. These analyses revealed that women do less housework and men do more housework in countries with higher levels of full‐time employment among women, greater provision of publicly funded child care, shorter maternal leaves, and more egalitarian gender attitudes.

However, the focus of much research using multilevel modeling has tended to be on the contribution of overall contextual effects relative to individual‐level effects across a large number of countries (necessary for the statistical assessment of contextual effects). This approach has two potential disadvantages. First, little space can usually be devoted to describing specific national policy contexts, or, more particularly, changes in those contexts. Second, it is also difficult to assess how disparate national policy contexts might be organized into more coherent policy‐related groupings. In contrast, more detailed comparisons of gendered time use in selected individual countries can provide more finely tuned information on national policy contexts. But, then again, this type of study, limited to comparisons of a few countries, can suffer from the difficulties of reconciling disparate national policy contexts into wider explanatory frames. I don't propose any answer to this—but I do think it is relevant to the fact that there has been more emphasis within the European sociological literature on the identification of meso‐level clusters of countries defined in terms of welfare policies and employment regimes.

Finally, within this section on methodological innovations, as a time‐use researcher I feel bound to point to some of the opportunities of time‐use data, and perhaps in particular to the significance of the time‐use data collected as part of the Harmonised European Time Use Survey (HETUS). Time‐use data record what I believe that Bronfenbrenner and Morris (1998) referred to as “micro‐time.” However, the accumulation of the different ways we spend our time is also a means by which our human capital and life chances develop—resonating also with the concept of meso‐time. To answer a direct critique made in the article, although it is undeniably true that the majority of time‐use research to date has focused on people in heterosexual couples, there is a growing body of research on other kinds of families, including same‐sex couples and nonpartnered individuals. There is also more attention being paid to the contributions of wider family structures and networks—for example, to the activities done and the enjoyment of time spent together with different family members.

Some time‐use diary surveys are better than others in providing the opportunity to conduct these more nuanced analyses. Although the American Time Use Survey (ATUS) collects time‐use information on activities from a single individual over a single day, the HETUS collects this information from entire households, including from children aged 8–12 and older, over 2 days (a weekday and a weekend day). In addition, individual questionnaires are conducted with all members of the HETUS households, collecting information on a wide range of individual‐level sociodemographic and economic characteristics and subjective measures such as general health, perception of being rushed, and activity preferences. They also collect detailed information on whom the respondent is providing help or care for, both within and without the household. This question is matched in the household questionnaire with a similar set of questions relating to whom the household is receiving any help or care from and for what purpose. The diary instrument itself also collects more information that is available in the ATUS, including multiple secondary activities over both days of the diary, and—in the French (2009–2010) and the new U.K. 2014–2015 HETUS—a field recording how much the respondent is enjoying what he or she is doing for every activity period over both days of the diary. These features contribute to the potential for some rich and varied analyses, including the sequence analysis of simultaneous trajectories of various household members' activities through the day. These developments move far beyond the more traditional individual‐level time‐budgeting approach that characterized early time‐use research. Furthermore, and referring back to the section on cross‐national research, this information is available in a standardized form across the countries of the European Union.

In addition, extra contextual information is increasingly being added to time‐use diary data, including, for example, information on the metabolic expenditure rates associated with different activities. Measures of environmental context, such as exposure to temperature and daylight (through adding daytime temperatures and sunrise and sunset times in the regions and season in which individual diaries were collected) may also go some way toward fulfilling some of the criteria necessary to contribute to the concept of macrotime. Methodological advances in time‐use data collection include the rapid and ongoing development of smartphone diary applications, making use of the GPS information available in these instruments; the measurement of actual individual energy expenditure by the use of accelerometers; and the use of wearable cameras that serve both to validate and to provide rich visual data to accompany the time‐use diary. This last innovation seems particularly relevant because it offers the opportunity to add a visual dimension to the sequences of activity generated by time‐use diary data, enabling actual observation of the daily processes and interactions involved in the production of family work and care.

My final set of comments relate to the issue of interdisciplinarity. Several things struck me in relation to this whilst reading the article. The ecological perspective is presented up front as the main framework of the article. But to what extent does the article—primarily a review—really reflect this? It seems to me that the focus might perhaps have been on a selection of studies from across academic disciplines that provide an application of the model rather than on all nominations for the Kanter Award? This might have helped me to understand better what the implications and data demands are of using such as perspective. For example, Tudge, Mokrova, Hatfield, and Karnik, (2009) provide a nice example of what a study using the person‐process‐context‐time (PPCT) model might look like in relation to child–parent interaction outcomes over time. I have to say that it's pretty demanding as a research description! But I believe that a combination of time‐use diaries attached to a panel study, as I have described, could potentially meet those demands.

In general, ecology does strike me as a slightly strange metaphor to have chosen to describe social structures. It is reminiscent of the way in which the “founding fathers” of sociology sought to equate social structures to biological or physical systems in an enterprise designed to enhance sociology's claims to scientific status (and their own claims to “grand theory!”). As a small example, I found the reference to ecological niches in the article difficult to interpret in terms of the literature that I normally read. I think the closest I could come would be social location, with a reference also to what I would understand by intersectionality. These differences in terminology of course result from different disciplinary perspectives, but as a sociologist I believe that choice of terminology is not neutral. As I perceive it, there is actually an important difference between an ecological niche and a social location, because an ecological niche may be frequently subject to entirely exogenous forces, such as the effects of human intervention unrelated to the organisms that inhabit that niche. I'm not certain that the same can be said about social locations (as determined by the intersectionalities of gender, race, social class, level of educational attainment, and so forth).

As for moving forward in an interdisciplinary way, although the article calls for more classificatory work to identify subdisciplines under the heading of work and family research, I would argue that an approach to synthesizing some of the elements of the ecological perspective with other multilevel theories might be a more synergistic suggestion. I am struck by the similarities between the ecological perspective as advanced by Bronfenbrenner and some recent multilevel processual perspectives from the sociological and feminist literatures. There is perhaps an opportunity missed here for an attempt at synthesizing, or at least addressing, the possible connections between an ecological perspective and other theoretical frameworks that also take as their foundation an integration of levels relating to individuals, contexts, and the processes that articulate them. Within a call for a more interdisciplinary approach to research on work and family, it seems to me that it would be worth exploring the connections between the ecological and these gender structure perspectives in particular. There were several examples appearing during the 1990s and 2000s of sociological models attempting to address the interwoven relationships between the levels of structure and action in relation to gender. A major contribution appeared with Risman's (1998, 2004) analysis of the gender structure, which, she writes, is a human invention and thus subject to change: “Actors shape the gender structure they inherit” (Risman, 1998, p. 5). Connell's (2000) concept of configurations of gender practice builds on the gender structure perspective, conceiving of masculinity and femininity as dynamic processes that have the ability to transform gender structures. My own model of embedded interaction describes a recursive process occurring across the levels of (a) individual resources and gender consciousness, (b) gendered interaction and negotiation, and (c) the wider discursive sphere. Within this model, daily interaction between individuals has a dialectic relationship with gender consciousness; is affected by the material and relational resources of each partner; and is (recursively) embedded within a wider discursive context (Sullivan, 2006). In a more recent joint reconceptualization with colleagues, we have proposed a longitudinal multilevel frame linking changes at the institutional and ideological levels to processes of change as they occur in interaction between men and women in the domestic sphere (termed *lagged generational change*). What is particularly pertinent in relation to the PPCT model is the explicit longitudinal dimension, a call for the examination of processes over time that is common to these multilevel theorizations. Of course, to actualize this longitudinal dimension in research, we need appropriate life‐course data (see above!).

To conclude, I think I can safely say that we are all in agreement that large‐scale social changes involve complex patterns of relations between institutional contextual factors, ideological structures, and individual‐level resources. I would love to see someone take on the relationship between concepts of the gender structure, actor–structure frameworks, life course analysis, intersectionality, and the ecological approach. This might lead to a truly interdisciplinary understanding of the relationships between structure and action, context and individual.
